# Dental Follicle Stem Cells Promote Periodontal Regeneration through Periostin-Mediated Macrophage Infiltration and Reprogramming in an Inflammatory Microenvironment

**DOI:** 10.3390/ijms24076353

**Published:** 2023-03-28

**Authors:** Xiuqun Wei, Shujuan Guo, Qian Liu, Li Liu, Fangjun Huo, Yafei Wu, Weidong Tian

**Affiliations:** 1 State Key Laboratory of Oral Diseases, & National Clinical Research Center for Oral Diseases, & National Engineering Laboratory for Oral Regenerative Medicine, West China School of Stomatology, Sichuan University, Chengdu 610041, China; 2 Engineering Research Center of Oral Translational Medicine, Ministry of Education, West China School of Stomatology, Sichuan University, Chengdu 610041, China; 3 Department of Periodontics, West China Hospital of Stomatology, Sichuan University, Chengdu 610041, China; 4 Department of Oral and Maxillofacial Surgery, West China Hospital of Stomatology, Sichuan University, Chengdu 610041, China

**Keywords:** periostin, dental follicle stem cells, macrophage reprogramming, periodontal regeneration

## Abstract

Dental follicle stem cells (DFSCs) have been verified to promote periodontal regeneration in an inflammatory microenvironment. When coping with inflammatory stimulation, DFSCs highly express periostin, a bioactive molecule closely related to periodontal homeostasis. It is worth exploring whether and how periostin plays a role in the promotion of periodontal regeneration by DFSCs. By tracking the fate of DFSCs, it was found that DFSCs significantly contributed to periodontal regeneration in rat periodontal defects while they had a low survival rate. They highly expressed periostin and improved the immune microenvironment in the defect area, especially via the recruitment and reprogramming of macrophages. Silencing periostin attenuated the effects of DFSCs in promoting periodontal regeneration and regulating macrophages. Recombinant human periostin (rhPeriostin) could not only directly promote macrophage reprogramming through the integrin αM/phosphorylated extracellular signal-regulated kinase (p-Erk)/Erk signaling pathway, but it also exhibited the potential to promote periodontal regeneration in rats when loaded in a collagen matrix. These results indicated that periostin is actively involved in the process by which DFSCs promote periodontal regeneration through the regulation of macrophages and is a promising molecular agent to promote periodontal regeneration. This study provides new insight into the mechanism by which DFSCs promote periodontal regeneration and suggests a new approach for periodontal regeneration therapy.

## 1. Introduction

Periodontitis is a highly prevalent infectious oral disease and reconstruction of periodontal tissue defects caused by periodontitis remains challenging [1,2]. A large number of animal and preclinical studies indicate that stem cell therapy is one of the most promising methods for reconstructing periodontal tissues [3,4,5,6,7] but there are still some unresolved problems, such as lack of relevant clinical data [8,9,10] and understanding the mechanism of action of stem cells [11].

Long-term chronic infection not only leads to destruction of tissue structure, but it also has adverse effects on the periodontal microenvironment, including disturbance of immune metabolism in the niche [12,13] and inhibition of endogenous stem cell function [14,15]. Studies have shown that the osteogenic differentiation ability of periodontal ligament stem cells (PDLSCs) is weakened in inflammation [15,16], which was not conducive to later repair. Therefore, it is necessary to find an effective method that can not only modulate locally damaged endogenous stem cells to restore osteogenic differentiation ability but also reprogram M1 to M2 macrophages to facilitate the subsidiary of periodontitis and subsequent regeneration. Our previous study showed that exosomes derived from *P. gingivalis* lipopolysaccharide (*P.g*-LPS)-preconditioned DFSCs enhanced the osteogenic differentiation of PDLSCs and promoted macrophage polarization [17], but the underlying mechanism remains extremely unclear.

Periostin is an extracellular matrix protein thought to be critical for building extracellular architecture [18] and has been reported to regulate cell–matrix interactions during tissue repair, inflammatory responses [19], and tumor metastasis [20,21]. It also plays an important role in the development and homeostasis of periodontal tissues [22]. The lack of periostin causes the destruction of periodontal tissues in mice [23,24]. Recent studies have proven that periostin could promote the proliferation, migration, and differentiation of periodontal-derived stem cells, showing the potential to promote periodontal regeneration [25,26,27,28]. In our previous study, it was found that DFSCs highly expressed periostin after inflammatory stimulation and could achieve better periodontal tissue regeneration than PDLSCs after implantation in the beagle canine periodontal defect model [16]. Whether periostin plays a role in the process of promoting periodontal regeneration by DFSCs and its mechanism are not yet clear.

In this study, we tracked the fate of DFSCs after transplantation into rat periodontal intrabony defects and explored the role and underlying mechanism of periostin in the promotion of periodontal regeneration by knocking down periostin in DFSCs. In addition, we investigated the therapeutic effect of using periostin as a molecular agent to promote periodontal regeneration. The null hypotheses were that periostin does not play a role in the promotion of periodontal regeneration by DFSCs and that application of periostin cannot promote periodontal regeneration. Our findings suggest that periostin mediates the process by which DFSCs promote periodontal regeneration by regulating macrophages via the integrin αM/p-Erk/Erk signaling pathway and that periostin has the potential to be used as a cell-free therapy to promote periodontal regeneration.

## 2. Results

### 2.1. DFSCs Significantly Promoted Periodontal Regeneration while the Cell Survival Rate Was Low after Transplantation

**Figure 1:**
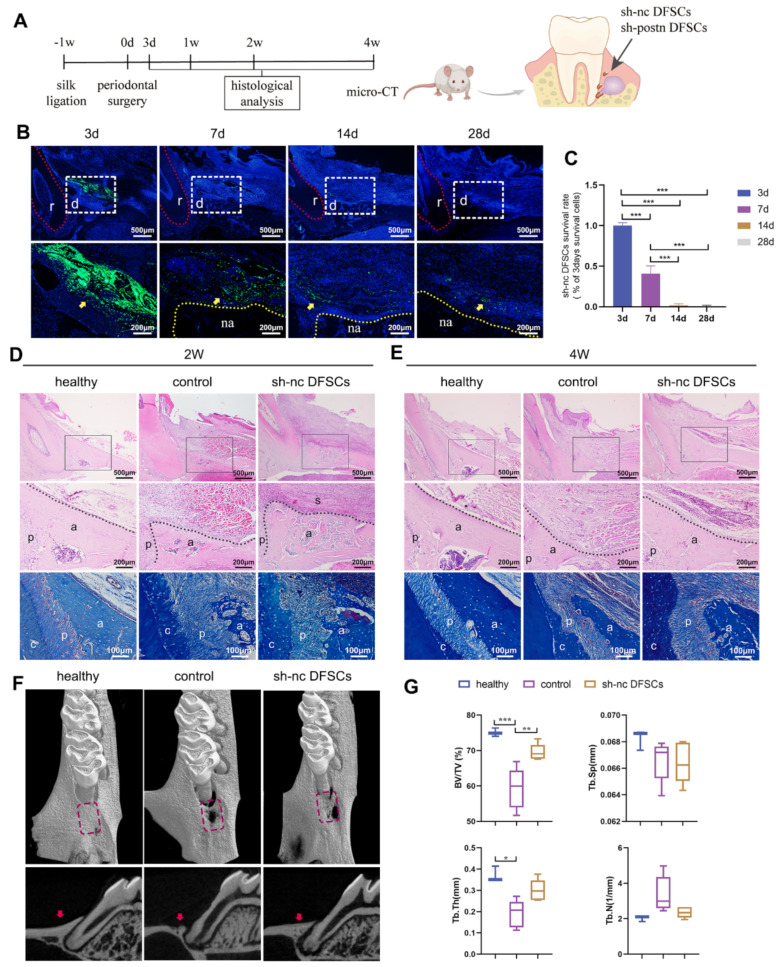
The therapeutic effect and survival rate of DFSCs. (**A**) Schematic illustration of animal model. Fluorescence images (**B**) and related analysis (**C**) of DFSC survival. The red dotted line indicates the root. The data were analyzed by one-way analysis of variance (ANOVA) and Bonferroni post hoc test (n = 3). The white dashed box indicates the defect area. The yellow dotted line indicates the alveolar bone. The yellow arrow indicates DFSCs. r: root; d: defect area; na: newly formed alveolar bone. HE and Masson’s trichrome (MT) staining at 2 weeks (**D**) and 4 weeks (**E**). Middle panel represents a magnified view of the black box in the upper panel. The black dotted line shows the newly formed alveolar bone. a: alveolar bone; c: cementum; p: periodontal ligament, s: DFSC sheet. Micro-computed tomography (micro-CT) images (**F**) and quantitative assessment (**G**) of the defect area at 4 weeks (n = 5). The data of Tb.Sp and Tb.N were analyzed by one-way ANOVA and Bonferroni post hoc test. The data of Tb.Th did not follow normal distribution and were analyzed by Kruskal-Wallis test. The rectangular area and red arrow indicate the defect area in the 3D and sagittal views, respectively. * *p* < 0.05; ** *p* < 0.01; *** *p* < 0.001. Error bars represent means ± SD.

The experimental periodontitis model in Sprague-Dawley (SD) rats was established at the first molars by the classical method of ligation and coating bacteria. One week later, a periodontal intrabony defect was created according to the previously described method [29,30] and transplanted with identified DFSCs (Figure 1A). The transplanted DFSCs were transfected with green fluorescent protein (GFP) lentivirus (sh-nc DFSCs) in advance to track their fate (Figure S1A). Three days after implantation, a large amount of green fluorescence could be observed in the defect area. At 7 days, approximately 40% of GFP+ transplanted cells were still located in the defect area, while at 2 and 4 weeks, they were greatly reduced to less than 5% (Figure 1B,C). In addition, GFP+ DFSCs did not co-localize with osteocytes, periodontal ligament cells, nor cementoblast cells but were always located around the regenerated periodontal tissues (Figure 1B).

At 2 weeks, newly formed bone tissue was observed in both the control and sh-nc DFSCs groups, but the latter was much better in terms of height and fullness (Figure 1D). In the sh-nc DFSCs group, the periodontal ligament was arranged in an orderly manner, while the arrangement was disordered in control group. At 4 weeks, the newly formed alveolar bone of the sh-nc DFSCs group was plump and dense, which was close to that of the healthy group (Figure 1E). The bone filling of the control group was significantly lower than that of the sh-nc DFSCs group; only a thin layer of alveolar bone could be observed on the surface of the root and the soft tissue in the defect area was loose and collapsed. In addition, the collapse of the gingival soft tissue was more obvious in the control group than in the sh-nc DFSCs group. The micro-CT assessment of alveolar bone at 4 weeks revealed significantly higher percentages of BV/TV in the sh-nc DFSCs group compared to that in the control group (Figure 1F,G). In addition, there was no statistical difference between the sh-nc DFSCs and healthy groups. These results suggested that despite a low survival rate after implantation, DFSCs promoted periodontal tissue regeneration in a rat model of inflammatory periodontal defect.

### 2.2. Transplanted DFSCs Suppressed Inflammatory Responses and Modulated Macrophage Infiltration and Polarization

**Figure 2:**
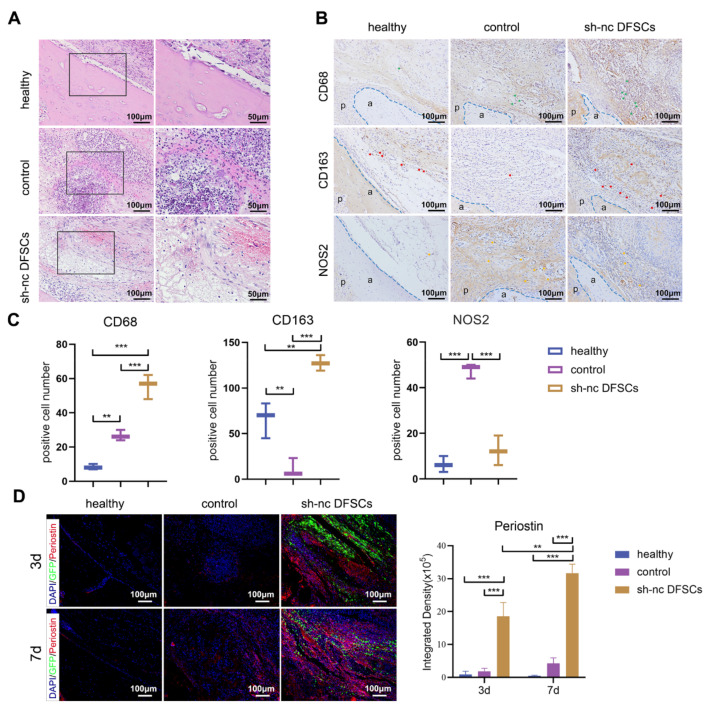
DFSCs improved the immune microenvironment and highly expressed periostin. (**A**) HE staining of the defect areas of the control and sh-nc DFSCs groups at 3 days after transplantation. The same area is shown in the healthy group. Right panel represents a magnified view of the black box in the left panel. The immunochemistry images (**B**) and quantification analysis (**C**) of the CD68+, CD163+, and NOS2+ cells on day 14 after surgery. The data were analyzed by one-way analysis of variance (ANOVA) and Bonferroni post hoc test (n = 3). The blue dotted line indicates the outline of the alveolar bone. a: alveolar bone; p: periodontal ligament. (**D**) Representative images of immunofluorescence staining against periostin in the defect area at 3 and 7 days. Data from 3d and 7d were analyzed independently by one-way analysis of variance (ANOVA) and Bonferroni post hoc test (n = 3). Data from the same treatment group at different time points were analyzed by unpaired two-tailed Student’s *t*-test (n = 3).; ** *p* < 0.01; *** *p* < 0.001. Error bars represent means ± SD.

The local immune microenvironment in the defect area was explored. At 3 days, the control group showed a typical acute inflammatory response with tissue congestion and edema and many inflammatory cells accompanied the defect areas. In the sh-nc DFSCs group, tissue edema was also seen but the infiltration of inflammatory cells was significantly less (Figure 2A). Macrophage infiltration was also examined. The sh-nc DFSCs group showed more CD68+ cells than the control group (Figure 2B,C). It is generally believed that the key to tissue repair is the proportion of M2 macrophages [31]. Hence, the numbers of M1 and M2 macrophages were assessed. Immunostaining against M2 marker CD163 revealed a larger amount of CD163+ cells in the sh-nc group than in the control group (Figure 2B,C). Additionally, the control group showed a higher number of inducible nitric oxide synthase (NOS2)+ macrophages (M1 macrophages) (Figure 2B,C). Taken together, DFSCs transplanted in the periodontal defect altered the local immune microenvironment, recruited macrophages, and increased their M2 polarization, thus promoting periodontal tissue regeneration in the inflammatory microenvironment. To investigate whether periostin participates in the process of DFSC regulation of immunity and promotion of periodontal regeneration, the expression level of periostin was examined, especially at the early repair stage. In the control group, the expression of periostin at 7 days was higher than that at 3 days. In the sh-nc group, no matter at 3 or 7 days, periostin showed a high level of expression, which was higher than that in the control group (Figure 2D). These results indicated that the excellent regeneration effects of DFSCs after implantation might be related to the high expression of periostin.

### 2.3. Silencing Periostin in DFSCs Prevented Macrophage Migration

**Figure 3:**
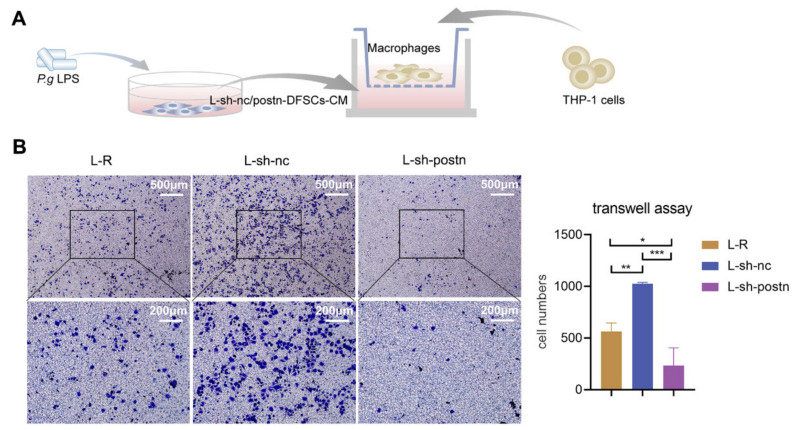
Silencing periostin in DFSCs prevented macrophage migration. (**A**) Schematic diagram to validate the effect of knocking down periostin on DFSC-promoted macrophage migration. (**B**) Representative images and quantitative analysis of macrophage migration under the DFSCs-CM. The data were analyzed by one-way ANOVA and Bonferroni post hoc test (n = 3). Images were taken at either 4× (upper row) or 10× (lower row) magnification. * *p* < 0.05; ** *p* < 0.01; *** *p* < 0.001. Error bars represent means ± SD.

The expression of periostin was knocked down in DFSCs to explore its role in the regulation of macrophages. *P.g*-LPS was used as described in previous research [16,32] to mimic an inflammatory microenvironment. The effect of silencing periostin in DFSCs on the regulation of macrophages, especially in recruiting macrophages, was examined. The conditioned medium from the supernatant of *P.g*-LPS-stimulated sh-nc DFSCs (sh-nc-CM) or sh-postn DFSCs (sh-postn-CM) was placed in the lower chamber, while the THP-1-derived macrophages were seeded in the upper chamber (Figure 3A). The addition of sh-nc-CM significantly promoted the migration of macrophages, while the addition of sh-postn-CM not only did not promote the migration of macrophages, but the number of migrated macrophages was even lower than that in the group without any addition of supernatant (Figure 3B). This finding suggested that periostin might play an important role in the biological functions of DFSCs in recruiting macrophages.

### 2.4. Silencing Periostin in DFSCs Inhibited the Promotion of Macrophage Reprogramming

**Figure 4:**
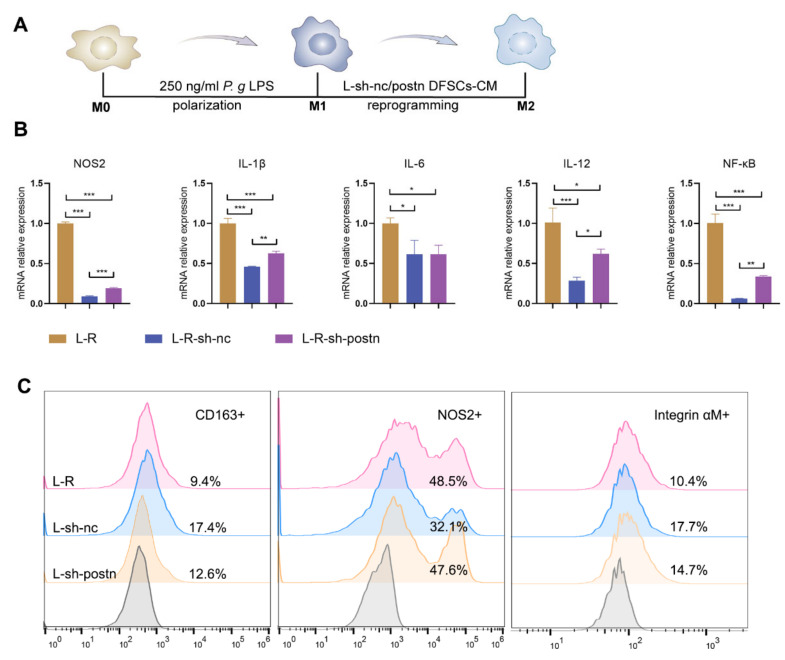
Silencing periostin inhibited the reprogramming of macrophages to the M2 type by DFSCs. (**A**) Diagram of the strategy for validating that periostin affects the ability of DFSCs to reprogram M1 to M2 macrophages. (**B**) qRT-PCR for M1 marker and pro-inflammatory factors in macrophages. The data were analyzed by one-way ANOVA and Bonferroni post hoc test (n = 3). (**C**) Flow cytometric analysis for surface marker expression on macrophages. * *p* < 0.05; ** *p* < 0.01; *** *p* < 0.001. Error bars represent means ± SD.

The immunomodulatory capacity of stem cells is not only manifested in recruiting macrophages but also in modulating the phenotype of macrophages. The mRNA expression levels of the M1 macrophage marker *NOS2* and pro-inflammatory factors interleukin (IL)-1β, IL-6, IL-12, and nuclear factor-kappa B (NF-κB) in the sh-nc DFSCs group were significantly lower than those in the control group. Although the mRNA expression levels of the M1 marker and pro-inflammatory factors in the sh-postn DFSCs group were still lower than those in the control group, they were higher than those in the sh-nc group (Figure 4B). Flow cytometry was used to detect changes at the cellular level. The percentage of CD163+ macrophages (M2 macrophages) in the sh-nc DFSCs group was significantly higher than that in the untreated group (L-R), while this regulatory ability was obvious weakened in the sh-postn DFSCs group. The treatment of sh-nc-CM significantly reduced the percentage of NOS2+ macrophages (M1 macrophages), while sh-postn-CM treatment was less effective (Figure 4C). These results suggested that silencing periostin inhibited the ability of DFSCs to promote the transformation of macrophages from the M1 type to the M2 type.

### 2.5. Silencing Periostin Impaired the Ability of DFSCs to Promote Periodontal Regeneration

**Figure 5:**
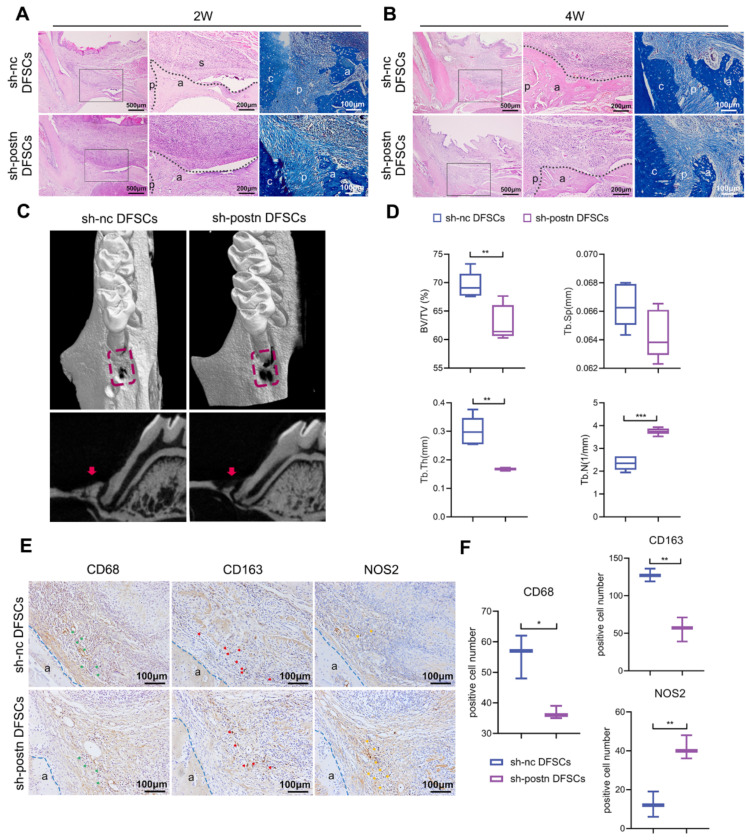
Silencing periostin affected the ability of DFSCs to promote periodontal regeneration. HE and MT staining of periodontal regeneration at 2 weeks (**A**) and 4 weeks (**B**). Middle panel represents a magnified view indicated by the black box in the left panel. The black dotted line shows the edge of the newly formed alveolar bone. a: alveolar bone; c: cementum; p: periodontal ligament; s: DFSCs sheet. Micro-CT images (**C**) and quantitative assessment (**D**) of the defect area at 4 weeks (n = 5). The red dotted box indicates the defect area and the red arrow indicates regenerated alveolar bone. The data of BV/TV, Tb.Sp, and Tb.N were analyzed by unpaired two-tailed Student’s *t* test. Unpaired two-tailed *t* tests with Welch correction were used for Tb.Th. The immunochemistry images (**E**) and quantification analysis (**F**) of the CD68+, CD163+, and NOS2+ cells on day 14 after surgery. green arrows: CD68+ cells; red arrows: CD163+ cells; yellow arrows: NOS2+ cells. The data were analyzed by unpaired two-tailed Student’s *t* test (n = 3). The blue dotted line indicates the outline of the alveolar bone. a: alveolar bone; p: periodontal ligament. * *p* < 0.05; ** *p* < 0.01; *** *p* < 0.001. Error bars represent means ± SD.

Then, the sh-nc and sh-postn DFSCs were cultured into cell sheets and transplanted into the periodontal defects, respectively. At 2 weeks, bone regeneration was observed in both groups, but the bone filling in the sh-postn DFSCs group was not as good as that in the sh-nc group (Figure 5A). In the sh-postn DFSCs group, the soft tissue in the defect area was loose and fragile, the collapse of the gingival soft tissue was more obvious, and the periodontal ligament was disordered (Figure 5A). At 4 weeks, the alveolar bone formed in both groups was much denser (Figure 5B). The bone filling and height of gingival attachment in the sh-postn DFSCs group were significantly lower than those in the sh-nc DFSCs group (Figure 5B). The arrangement of the periodontal ligament in both groups was parallel and orderly (Figure 5B). The micro-CT analysis showed that the BV/TV and Tb.Th of the sh-postn DFSCs group were significantly lower than those of the sh-nc DFSCs group (Figure 5C,D). Conversely, the Tb.N of the sh-postn DFSCs group was higher than that of the sh-nc DFSCs group.

The local immune microenvironment in the defect area was explored. The infiltration of macrophages in the sh-postn DFSCs group was less than that in the sh-nc DFSCs group (Figure 5E,F). It is generally believed that the key to tissue repair is the proportion of M2 macrophages [31]. Hence, the numbers of M1 and M2 macrophages were assessed. The number of CD163+ M2 macrophages in the sh-postn DFSCs group was also lower than that in the sh-nc DFSCs group, while the number of NOS2+ M1 macrophages in the sh-postn DFSCs group was higher (Figure 5E,F). These results demonstrated that endogenous inhibition of periostin impaired the ability of the DFSC sheet to modulate macrophage polarization and promote periodontal tissue regeneration in vivo.

### 2.6. Periostin Reprogrammed M1 to M2 Macrophages in Part through Integrin αM/p-Erk/Erk Signaling

**Figure 6:**
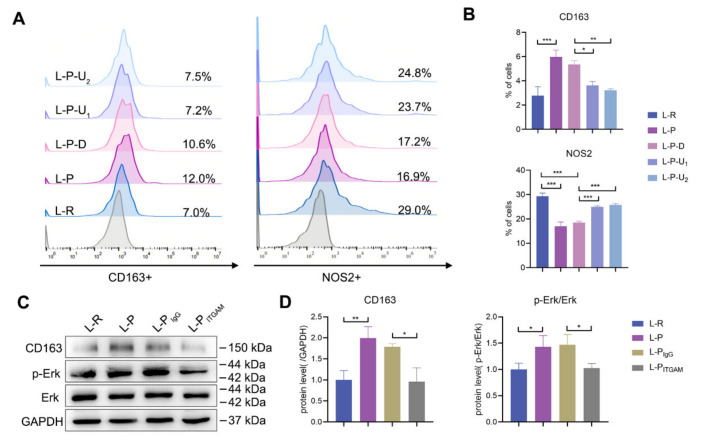
Periostin reprogrammed macrophages partly through integrin αM/p-Erk/Erk signaling. (**A**,**B**) Flow cytometric analysis for pro- and anti-inflammatory marker expression in macrophages after periostin stimulation with or without Erk inhibitor. The data were analyzed by one-way ANOVA and Bonferroni post hoc test (n = 3). L-R: group treated with RPMI1640 alone; L-P: group treated with rhPeriostin; L-P-D: group treated with rhPeriostin and DMSO; L-P-U_1_: group treated with rhPeriostin and 10 μg/mL U0126; L-P-U_2_: group treated with rhPeriostin and 20 μg/mL U0126. (**C**,**D**) Western blot analysis of CD163 and phosphorylation of ERK in macrophages after periostin stimulation with or without anti-ITGAM antibodies. IgG isotype control or anti-ITGAM antibodies were added to examine the role of integrin αM in signaling. The data were analyzed by one-way ANOVA and Bonferroni post hoc test (n = 3). * *p* < 0.05; ** *p* < 0.01; *** *p* < 0.001. Error bars represent means ± SD.

The most obvious difference between sh-nc and sh-postn DFSCs was the expression of periostin. Therefore, rhPeriostin was used to treat the macrophages and verify the direct regulatory effect of periostin on the macrophages. Previous studies have shown that the Erk signaling pathway is closely related to M2-type polarization of macrophages [17,33]. Therefore, an inhibitor of the Erk signaling pathway (U0126) was added to further explore the mechanism. RhPeriostin promoted the percentage of M2 macrophages and reduced the percentage of M1 macrophages (Figure 6A,B). When the inhibitor of the Erk signaling pathway was added, this regulatory effect was significantly inhibited (Figure 6A,B). These results indicated that the promoting effect of rhPeriostin on M2 macrophages was partly through the activation of its downstream p-Erk/Erk signaling pathway.

Periostin is an extracellular matrix protein and its main way of acting is to bind to cell surface integrin receptors [18]. We also observed the differential activation of integrin αM in macrophages when they were treated with sh-nc DFSC-CM and sh-postn DFSC-CM (Figure 4C). Next, the neutralizing antibody of integrin αM was used. Western blotting showed that p-Erk/Erk signaling was inhibited and the expression of CD163 was decreased (Figure 6C,D). These results suggested that rhPeriostin promoted the expression of CD163 in the inflammatory microenvironment mainly by binding to the macrophage surface receptor integrin αM and activating the downstream p-Erk/Erk signaling pathway.

### 2.7. Periostin–Collagen Matrix Promoted Periodontal Regeneration

**Figure 7:**
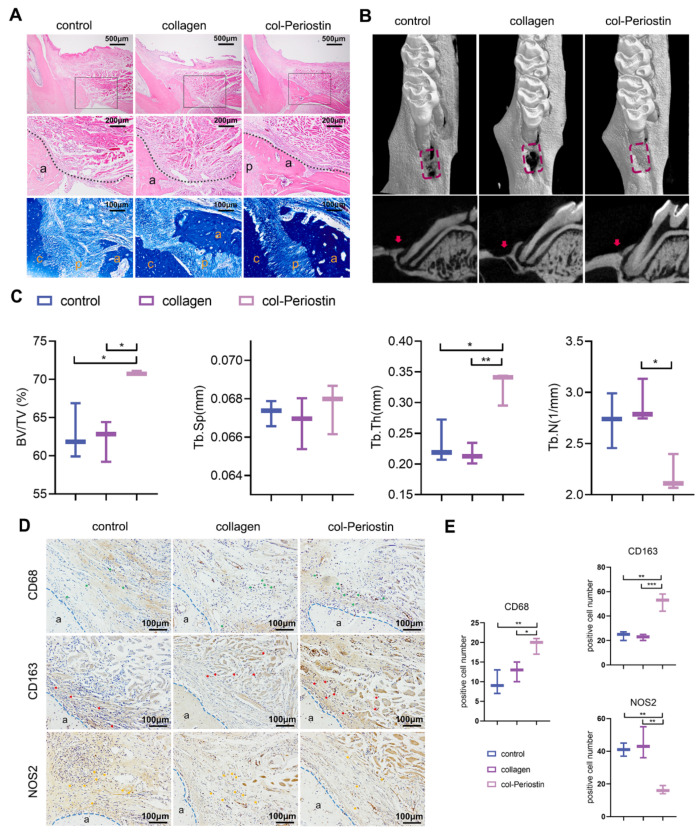
RhPeriostin promoted periodontal tissue regeneration. (**A**) HE and MT staining of periodontal regeneration at 4 weeks after periostin treatment. Images shown are representative images. Middle panel represents a magnified view of the black box in the upper panel. The black dotted line shows the edge of the newly formed alveolar bone. a: alveolar bone; c: cementum; p: periodontal ligament. Micro-CT images (**B**) and quantitative assessment (**C**) of the defect area at 4 weeks (n = 3). The red dotted box indicates the defect area, and the red arrow indicates regenerated alveolar bone. The data of BV/TV, Tb.Sp, Tb.N, and Tb.Th were analyzed by one-way ANOVA and Bonferroni post hoc test. The immunochemistry images (**D**) and quantification analysis (**E**) of the CD68+, CD163+, and NOS2+ cells on day 28 after surgery. green arrows: CD68+ cells; red arrows: CD163+ cells; yellow arrows: NOS2+ cells. The data were analyzed by one-way ANOVA and Bonferroni post hoc test (n = 3). The blue dotted line indicates the outline of the alveolar bone. a: alveolar bone. * *p* < 0.05; ** *p* < 0.01; *** *p* < 0.001. Error bars represent means ± SD.

Although the results of the in vitro experiments showed the ability of periostin in converting macrophages from a pro-inflammatory phenotype to an anti-inflammatory and pro-regenerative phenotype, it was still unknown whether periostin plays a role in a very complex microenvironment such as periodontal tissues. As a key factor in the process of promoting periodontal tissue regeneration by DFSCs, periostin may provide a substitute for DFSCs for better clinical application if it can achieve the desired regeneration effect alone. Here, for the convenience of application, a collagen gel was chosen as the material for loading periostin. Then, the mixture (rhPeriostin + collagen gel, col-Periostin) was injected into the periodontal defect area. Groups were also treated with the collagen gel (collagen) or nothing (control). The results of histological staining at 4 weeks showed that new alveolar bone could be observed in the three groups, but the bone filling of the col-Periostin group was significantly better than that of the other two groups and the alveolar bone was denser, similar to the healthy group (Figure 7A). Micro-CT analysis showed that the BV/TV and Tb.Th in the col-Periostin group were significantly higher than those in collagen and control groups (Figure 7B,C). The Tb.N in the col-Periostin group was significantly lower than that in the collagen group. There were no statistical differences among the three groups for Tb.Sp. These results demonstrated the effect of periostin in promoting periodontal tissue regeneration.

The infiltration and polarization of macrophages in the defect area were also explored. The results of immunohistochemistry showed that the numbers of CD68+ macrophages and CD163+ M2 macrophages in the col-Periostin group were significantly higher than those in the other two groups (Figure 7D,E). The number of NOS2+ M1 macrophages in the col-Periostin group was lower than that in the other two groups (Figure 7D,E). These results indicated that col-Periostin treatment significantly improved the immune microenvironment in the defect area.

## 3. Discussion

**Figure 8:**
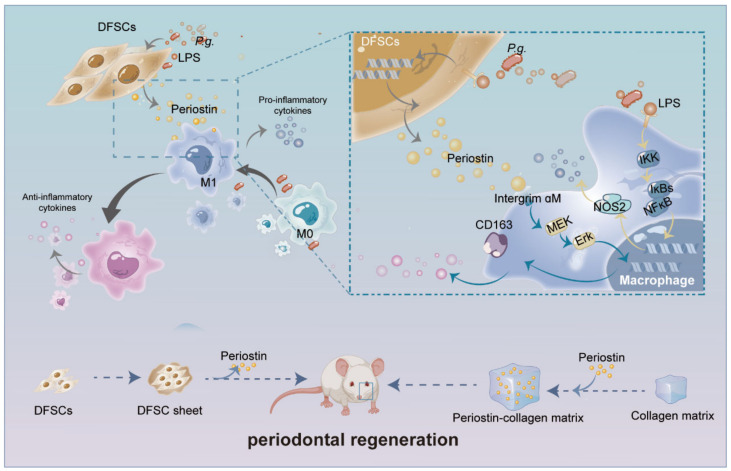
Schematic diagram of the role and mechanism of periostin in the promotion of periodontal regeneration by DFSCs. Periostin is actively involved in DFSC-mediated periodontal regeneration through regulating macrophages via integrin αM/p-Erk/Erk signaling and is a promising molecular agent to promote periodontal regeneration.

This study demonstrated that DFSCs had a low survival rate after transplantation, and periostin was actively involved in the process of DFSCs regulating macrophages to promote periodontal regeneration. It was also found that periostin could reprogram macrophages through the integrin αM/p-Erk/Erk signaling pathway. Implantation of a collagen matrix-loaded rhPeriostin promoted periodontal regeneration in inflammatory periodontal defects in rats (Figure 8).

DFSCs are one of the candidate cells for promoting periodontal tissue regeneration [34,35]. They promoted periodontal regeneration even in an inflammatory microenvironment caused by long-term chronic infection of periodontal tissues [16]. However, the fate of DFSCs after transplantation in infected periodontal defects and the underlying mechanism by which DFSCs promote periodontal regeneration were unknown. In the present study, the transplanted DFSCs were monitored for a period of 28 days, which showed that the number of surviving DFSCs decreased over time. Interestingly, despite experiencing rapid death during the first two weeks, a part of the transplanted DFSCs were still able to be detected in the defect area at 28 days. Whether these cells eventually “disappear” over time or become permanent residents remains a mystery. A previous study showed that implanted periodontal ligament cells could no longer be detected in the periodontal defect 10 weeks after transplantation [36]. However, the temporal and spatial fates of the cells that “disappear” after transplantation need to be revealed.

Additionally, attention should be paid to whether the transplanted DFSCs differentiate in the defect. An earlier study reported differentiation of transplanted MSCs in the regenerated periodontal tissues [37]. However, follow-up studies showed that the transplanted MSCs were not integrated into the newly formed periodontal tissues [36]. Even though these transplanted cells partially differentiated, their contribution to the newly formed periodontal tissues was minimal [38,39,40]. In the present study, the differentiation of DFSCs was also not observed. The transplanted DFSCs did not integrate into the newly formed periodontal tissues but were always located around the regenerated tissues. Taken together, these results suggest that most transplanted DFSCs are likely sacrificed in vivo and rarely differentiate.

It is likely that the implanted DFSCs induce pro-regenerative effects by means of indirect effects, without generating new bone tissues themselves. Macrophages are closely related to tissue regeneration [41,42,43]. Our study also observed changes in the local immune microenvironment in the defect after transplantation of DFSCs, especially an increase in M2 macrophages. The phenotype of macrophages affects not only the development of periodontitis but also the repair and regeneration of periodontal tissues [44,45,46,47]. Previous studies have shown that DFSCs could act on macrophages by secreting soluble factors, resulting in the transformation of M1 into M2 macrophages in the inflammatory microenvironment [17,48]. However, it is not known exactly which kinds of active molecules play a role. Our previous study observed that DFSCs highly expressed periostin after *P.g*-LPS induction [16]. In this study, the expression of periostin was also examined in the inflammatory periodontal defects after DFSC implantation. The transplanted group showed persistently high expression of periostin during the early repair stage, which suggested that periostin might play a role in the regulatory effect of DFSCs. In fact, several recent studies have gradually reported the role of periostin in regulating macrophages in other diseases, such as glioblastoma [49,50], spinal cord injury [51], and ovarian cancer [52]. In a mouse model of myocardial infarction, injected cardiac resident stem cells promoted myocardial regeneration by regulating the M2-type polarization of macrophages through periostin [53]. Therefore, the periostin gene was silenced in DFSCs to verify the role of periostin in DFSC regulation of macrophages. The results showed that periostin silencing impaired the ability of DFSCs to regulate macrophages in an inflammatory microenvironment, specifically in reducing macrophage infiltration and inhibiting macrophage transformation to the M2 type.

Periostin can also directly promote the M2 polarization of macrophages, but its intracellular mechanism is not yet clear. Previous studies have shown that the Erk signaling pathway is closely related to M2-type polarization of macrophages [17,33,54]. When the Erk signaling pathway inhibitor was added, the effect of periostin in promoting M2 macrophage polarization was reversed. These results suggested that the role of periostin in promoting M2-type polarization of macrophages is generated through the Erk signaling pathway.

Periostin is an extracellular matrix protein. The main way that periostin exerts its biological effects is to bind to the integrin family receptors on the surface of the cell membrane, thereby triggering biological changes in the cell [55]. This study confirmed the targeted binding of periostin to integrin αM on the macrophage surface and activation of the downstream Erk signaling pathways by using an antagonist of integrin αM. This result was consistent with a recent study in which periostin was reported to promote immune cell survival by binding to integrin αM [56]. Notably, the use of integrin αM antagonists only partially altered the Erk signaling pathway. This may be because periostin can bind to a variety of cell surface receptors, such as integrin αvβ3 in periodontal ligament cells [57].

DFSCs have showed considerable advantages in promoting periodontal tissue regeneration [16,34,58]. However, there are still many difficulties in the clinical application of stem cells, especially possible utility, safety, and ethical issues [59]. Molecular drugs may be relatively more convenient, safe, and stable [60,61]. The concept of endogenous regeneration holds that the therapeutic potential of endogenous stem cells can be harnessed by using bioactive molecules and material matrices, thereby unleashing the innate power of the body and promoting in situ tissue regeneration [62]. From exploring the mechanism by which DFSCs promote periodontal regeneration, an important bioactive molecule, periostin, was found and shown to actively participate in the regulation of macrophage phenotype transformation through the integrin αM/p-Erk/Erk signaling pathway. In addition, periostin can also induce the homing and differentiation of periodontal endogenous stem cells [25,63]. Thus, periostin was mixed with collagen gel and applied to infected periodontal defects to observe its effect on promoting periodontal tissue regeneration. The results preliminarily confirmed that periostin could promote periodontal regeneration. Notably, we also found that the promotion of tissue regeneration produced by the combination of periostin plus collagen gel was the same as that obtained with DFSCs. This demonstrated that periostin is expected to become a candidate molecular drug for clinical use. This study is the first attempt to the translational application of periostin in periodontal tissues; more standard and reliable preclinical and clinical experiments are still indispensable to further prove its safety and efficacy.

However, there are still some limitations in this study. The animal sample size was not large enough, which may have caused sample bias. It is necessary to expand the sample size in further research. In addition, the loading system in this study was relatively simple. In order to conveniently apply rhPeriostin to periodontal defects, a commercialized collagen gel was chosen as the carrier. Subsequent research will design a more suitable carrier with slow- and controlled-release functions for this protein.

## 4. Materials and Methods

### 4.1. DFSC Isolation and Culture

The isolation and culture of DFSCs was previously described [64]. *P.g-*LPS (Invivogen, San Diego, CA, USA) at 250 ng/mL was used to stimulate DFSCs in order to mimic the inflammatory microenvironment, and DFSC sheets were obtained as previously described for in vivo application [16]. Briefly, DFSCs were seeded in 24-well plates at 5 × 10^4^ per well. Cell sheets were cultured for 14 days using α-MEM medium (Hyclone, Logan City, UT, USA) containing 10% FBS, 100 U/mL penicillin/streptomycin, and 50 μg/mL vitamin C. The area of the cell sheet used for transplantation was consistent with the area of a 24-well plate bottom (2 cm^2^). All experiments with DFSCs were conducted with ethical approval from the Committee of Ethics of Sichuan University, and written informed consent was obtained from all guardians on behalf of the children and teenagers enrolled in this study. The approval number is WCHSIRB-CT-2021-509.

### 4.2. Lentivirus Transfection

The design and synthesis of the sh-nc and sh-postn lentiviruses were completed by Hanheng Biotechnology. DFSCs were infected with sh-nc and sh-postn lentiviruses according to the manufacturer’s instructions. Briefly, DFSCs were plated overnight and then infected with lentiviruses at a multiplicity of infection (MOI) of 30 in the presence of polybrene (5 μg/mL; Sigma-Aldrich, St. Louis, MO, USA) for 48 h. After 48 h, infected cells were selected with 1 μg/mL puromycin (Sigma-Aldrich, St. Louis, MO, USA) for 48 h. Cells at the 3rd–5th passages were used for subsequent experiments.

### 4.3. Preparation of DFSC-Conditioned Medium

2 × 10^5^ sh-nc and sh-postn DFSCs were inoculated into 6-well plates, respectively. When sh-nc and sh-postn DFSCs reached certain confluence (60–70%), the medium was changed to fresh RPMI 1640 medium (Hyclone, Logan City, UT, USA) supplemented with 10% FBS, 100 U/mL penicillin/streptomycin, and 250 ng/mL *P.g*-LPS and harvested after 24 h of culture. The collected medium was centrifuged at 3000 rounds/min for 10 min to remove debris and was used as the conditioned medium of sh-nc and sh-postn DFSCs (sh-nc DFSC-CM and sh-postn DFSC-CM).

### 4.4. THP-1 Cell Culture

Human acute leukemia monocytic cell line THP-1 was purchased from the American Type Culture Collection (ATCC). Cells were seeded in 6-well plates (2 × 10^6^ cells/well) and cultured in RPMI 1640 medium (2 mL/well) supplemented with 10% FBS and 100 U/mL penicillin/streptomycin. Cells from passages 4 to 10 were used. To generate macrophages, monocytes were treated with 100 ng/mL phorbol 12-myristate-13-acetate (PMA, San Diego, CA, USA) for 48 h. To polarize into M1 macrophages, macrophages were further treated with 250 ng/mL of *P.g*-LPS for 24 h. Then, half of the old medium (1 mL) was removed and replaced with the same volume of sh-nc DFSC-CM or sh-postn DFSC-CM (delete or rhPeriostin [R&D Systems, Minneapolis, MN, USA] was used to treat M1 macrophages) (Figure 4A). After 24 h, cells were collected for subsequent analysis.

In the same manner, 100 ng/mL rhPeriostin in RPMI1640 medium (1 mL) or medium alone was used to treat *P.g*-LPS-stimulated macrophages in order to explore the direct role of periostin in regulating macrophage polarization. The rhPeriostin concentration was determined according to the preliminary experiment results. 10 or 20 μM U0126 (Selleck Chemicals, Houston, TX, USA) was co-treated with rhPeriostin to block the Erk signaling pathway. DMSO was used as a solvent control. After 24 h, cells were collected for subsequent analysis.

The anti-ITGAM antibody (eBioscience, San Diego, CA, USA) was also co-treated with rhPeriostin at 10 μg/mL for 24 h. Mouse IgG isotype control antibody (eBioscience, San Diego, CA, USA) was used as an isotype control.

### 4.5. Transwell Assay

Macrophages was seeded at 1 × 10^6^ cells/mL into the upper chamber in a total volume of 250 μL. Transwell inserts with 8 μm pores (Corning, NY, USA) were used. Sh-nc DFSC-CM or sh-postn DFSC-CM was added to RPMI 1640 containing 250 ng/mL *P.g-*LPS at a 1:1 ratio (500 µL total) to the lower chamber. The group to which only *P.g-*LPS-containing RPMI 1640 was added was used as the control. Macrophages were allowed to migrate through the transwell insert membrane for 24 h. The inserts were removed, fixed in cold methanol, and stained with methanol containing 1% crystal violet. The inside of the Transwell was swabbed thoroughly with cotton swabs and air dried at room temperature overnight. The number of migrated macrophages was counted by bright-field microscopy at 4× and 10×. Five bright-field images were analyzed per well.

### 4.6. Quantitative Reverse Transcription–Polymerase Chain Reaction (qRT–PCR)

Total RNA was isolated using TRIzol reagent according to the manufacturer’s protocol (TIANMO, Beijing, China). RNA (1 µg) was converted into cDNA using an iScript cDNA synthesis kit (Vazyme, Nanjing, China). Gene expression was quantified by the 2^−ΔΔCt^ method using glyceraldehyde 3-phosphate dehydrogenase (GAPDH) expression as an internal control. The primers used for target genes are listed in Supplementary Table S1.

### 4.7. Flow Cytometry

The expression of integrin αM, CD163, and NOS2 in THP-1-derived macrophages was assayed via fluorescence using the BD Accuri C6 (Becton Dickinson, San Jose, CA, USA) and analyzed using FlowJo V10 software. The adherent cells were collected and stained with antibodies for 30 min at 4 °C according to the manufacturer’s instructions. The antibodies used are listed in Supplementary Table S2.

### 4.8. Western Blot

Cells were lysed in RIPA buffer (KeyGEN Biotech, Nanjing, China) with 1 mM proteinase inhibitor phenylmethanesulfonylfluoride and 1% phosphatase-inhibitor (Sigma-Aldrich, St. Louis, MO, USA) for 30 min, and then the protein was extracted. The antibodies used are listed in Supplementary Table S2. The relative intensity of the tested protein was quantitatively analyzed by the ratio of the gray value between the target protein and GAPDH in the same sample.

### 4.9. Animals

Animals were obtained from Dashuo Experimental Animal Co., Ltd. (Chengdu, China). This study was reviewed and approved by the Ethics Committees of the State Key Laboratory of Oral Diseases, West China School of Stomatology, Sichuan University. The approval number is WCHSIRB-D-2021-555. The care and use of the laboratory animals followed the guidelines of the Institutional Animal Care and Use Committee of West China School of Stomatology, Sichuan University. All animals were kept in a controlled environment (50% humidity, 25 °C, and 12 h light–dark cycle) with free access to food and water. All the animal experiments were carried out at the animal center of Sichuan University and the State Key Laboratory of Oral Diseases, West China School of Stomatology, Sichuan University.

After one week of acclimation, 87 healthy eight-week-old male SD rats that met the inclusion and exclusion criteria were randomized into groups using Microsoft Excel. The inclusion and exclusion criteria were established a priori and are provided in Supplementary Table S3. In the animal experiments to explore the therapeutic effect and mechanism by which DFSCs promote periodontal regeneration, there were three groups: healthy group (no surgery and no treatment, n = 12, n = 3 per time point); control group (underwent surgery but not treated, n = 20, n = 5 per time point); sh-nc DFSCs group (underwent surgery and implanted with sh-nc DFSC sheet, n = 20, n = 5 per time point). In the animal experiments to explore the role of periostin in the promotion of periodontal regeneration by DFSCs, there were two groups: sh-nc DFSCs group (n = 10, n = 5 per time point); sh-postn DFSCs group (underwent surgery and implanted with sh-postn DFSC sheet, n = 10, n = 5 per time point). In the animal experiments to explore the therapeutic effect of periostin in periodontal regeneration, there were three groups: control group (n = 5); collagen group (underwent surgery and implanted with collagen gel, n = 5); col-Periostin group (underwent surgery and implanted with rhPeriostin-loaded collagen gel, n = 5). The number of SD rats per experimental group was determined based on our previous experience with similar models [65].

Blinding was used at all stages of the animal experiments whenever possible, including allocation, surgery and treatment, outcome assessment, and data analysis. For each animal, three different investigators were involved as follows: The first investigator performed the surgery and administered treatment according to the randomization table. This investigator was the only person who knew the group allocation. A second investigator was responsible for sample collection and processing. Finally, a third investigator performed the data analysis.

### 4.10. Rat Periodontal Defect Model

The rat periodontal defect model used in this experiment referred to previous studies [29,30] and was improved according to the actual situation of this study. Rats were anesthetized with pentobarbital (1 mg/kg) via intraperitoneal injection and pain was minimized with buprenorphine (0.05 mg/kg). The healthy group was not treated and the periodontitis model was established by ligaturing with 5.0 cotton threads and inoculating *P.g* (ATCC 33277, 2 × 10^9^ colony forming unit/mL; Chengdu, China) at the left upper jaw first molar for 1 week. Then, periodontal defects of size 1.5 × 2 × 1 mm (W × L × D) were created on the mesial surface of the first molar. The defect was then rinsed with saline and dried with sterile cotton pellets to ensure hemostasis was achieved before it was left untreated (control) or implanted with sh-nc DFSC sheets (sh-nc DFSCs), sh-postn DFSC sheets (sh-postn DFSCs), rhPeriostin-loaded collagen gel (col-Periostin), or control collagen gel (collagen). Finally, the flap was closed with single interrupted sutures (Vicryl 6-0, Ethicon Products, Amersfoort, The Netherlands) and primary closure was achieved. Subcutaneous injections of antibiotics (Eufloxacin; 0.1 mL/100 g) and analgesics (Camprofen; 0.1 mL/100 g) were given once daily for 3 days post-operatively. At 3, 7, 14, and 28 days post-surgery, animals were euthanized with sodium pentobarbital (150 mg/kg) by intraperitoneal injection for subsequent analysis in animal experiments to explore the therapeutic effect and mechanism by which DFSCs promote periodontal regeneration. In the animal experiments to verify the role of periostin in the promotion of periodontal regeneration by DFSCs, animals were euthanized on days 14 and 28 post-operation. In the animal experiments to explore the therapeutic effect of periostin in periodontal regeneration, animals were euthanized on day 28 post-operation. The individual rat was considered the experimental unit. All animals surviving the experimental treatment were included in the final analysis.

### 4.11. Preparation and Use of Periostin–Collagen Matrix

The reconstituted collagen solution was prepared according to the instructions (Nitta Gelatin, Osaka, Japan). Then, an appropriate amount of rhPeriostin was added and mixed to make the final concentration 100 ng/mL. The solution was aliquoted by the defect volume and placed at 37 °C for 30 min to allow the solution to turn into a gel. The above gels were implanted into the maxillary first molar defects of rats.

### 4.12. Micro-Computed Tomography Evaluation

The harvested and fixed maxillas was scanned by a SkyScan 1176 desktop X-ray micro-CT system (Skyscan, Kontich, Belgium) at 18 μm resolution. According to the size and location of the defect, a region of interest (ROI) of approximately 3 mm^3^ was defined for the 3-dimensional (3-D) measurements and analysis. The percentage of bone volume over total volume (BV/TV, %), trabecular separation (Tb.Sp, mm), trabecular thickness (Tb.Th, mm), and trabecular number (Tb.N, 1/mm) were analyzed by CTAn software (Burker micro-CT, Kontich, Belgium). The primary outcome measure was BV/TV.

### 4.13. Histological, Immunohistochemical, and Immunofluorescent Analyses

All bone specimens were decalcified, dehydrated, and embedded. Serial sections (5 µm) were obtained and then analyzed with hematoxylin and eosin (HE) staining, Masson’s trichrome (MT) staining, immunohistochemistry, and immunofluorescence according to the manufacturers’ recommended protocol. The antibodies used are listed in Supplementary Table S2. All samples were imaged under an inverted microscope (Olympus, Japan) or confocal laser scanning microscopy (Olympus, Tokyo, Japan) and semi-quantitatively analyzed using Image J software. Each assay was run in triplicate. In the immunohistochemical experiments, three fields of view were randomly selected for each slice and the number of macrophages in the ROI (defect area) was manually counted using ImageJ. In the immunofluorescent analysis, three fields of view were randomly selected for each slice and the fluorescence intensity in the ROI was measured using ImageJ. Slices from at least three independent experiments per group were selected for statistical analysis.

### 4.14. Statistical Analysis

All data were analyzed using GraphPad Prism 8.0.2 software and presented as the mean ± standard deviation (mean ± SD). The confidence interval was 95%. All data were tested for normality using the Shapiro–Wilk test. The unpaired two-tailed Student’s *t* test was used for comparison between two groups of data with normal distribution and homogeneous variance. Welch’s correction was used when the data did not meet the homogeneity of variance. The Mann–Whitney U test was used to compare two groups of data that did not follow normal distribution. One-way analysis of variance (ANOVA) and Bonferroni post hoc test were used to compare three or more groups of data with normal distribution and homogeneous variance. If the data did not follow normal distribution, the Kruskal-Wallis test was used. *p* < 0.05 was considered statistically significant.

## 5. Conclusions

Taken together, these data demonstrate that periostin is actively involved in the process by which DFSCs promote periodontal regeneration through regulation of the periodontal immune microenvironment and might provide a promising molecular agent to promote periodontal regeneration.

## Data Availability

The data are not publicly available due to privacy or ethical restrictions. The data are not publicly available due to privacy or ethical restrictions.

## Supplementary Materials

The following supporting information can be downloaded at: https://www.mdpi.com/article/10.3390/ijms24076353/s1.

## Abbreviations

DFSCs: Dental follicle stem cells; GFP: Green fluorescent protein; SD: SD: Sprague–Dawley; RhPeriostin: Recombinant human Periostin; *P.g*: *P. gingivalis*; LPS: Lipopolysaccharide; α-MEM: Alpha modification of Eagle’s Medium; sh-nc DFSC-CM: Conditioned medium of sh-nc DFSCs; sh-postn DFSC-CM: Conditioned medium of sh-postn DFSCs; MOI: Multiplicity of infection; PMA: Phorbol 12-myristate-13-acetate; qRT-PCR: Quantitative reverse transcription–polymerase chain reaction; ROI: Region of interest; HE: Hematoxylin and eosin, MT: Masson’s trichrome; IHC: Immunohistochemical; Micro-CT: Micro-computed tomography; BV: Bone volume; TV: Total volume; Tb.Sp: Trabecular separation; Tb.Th: Trabecular thickness; Tb.N: Trabecular number; GAPDH: Glyceraldehyde 3-phosphate dehydrogenase; Erk: Extracellular signal-regulated kinase; IL: Interleukin; NOS2: Inducible nitric oxide synthase; NF-κB: Nuclear factor-kappa B.
