# Supplementary material for: Dental Follicle Stem Cells Promote Periodontal Regeneration through Periostin-Mediated Macrophage Infiltration and Reprogramming in an Inflammatory Microenvironment
Source: Int J Mol Sci. 2023 Mar 28;24(7):6353. doi: 10.3390/ijms24076353 (PMC10094259; doi:10.3390/ijms24076353)
Supplement: Supplementary file 1 [file ijms-24-06353-s001.zip › ijms-2259374-supplementary.pdf]

Supplementary information and results

Table S1. Primer sequence used for quantitative RT-PCR

Gene	Forward primer	Reverse primer
human Periostin	5'-CACTCTTTGCTCCCACCAATA-3'	5'-ATTTCCTTCCAGCGTCTCAA-3'
human NOS2	5'-TTTCCAAGACACACTTCACCA-3'	5'-ATCTCCTTTGTTACCGCTTCC-3'
human IL-1β	5'-CTTTGAAGCTGATGGCCCTAAA-3'	5'-AGTGGTGGTCGGAGATTCGT-3'
human IL-6	5'-TGTGAAAGCAGCAAAGAGGC-3'	5'-TGGGTCAGGGGTGGTTATT-3'
human IL-12	5'- TTCCCTGGTTTTTCTGGCATCTC-3'	5'-CTTGGATGGTCAGGGTTTTGCCA-3'
human NF-κB	5'-GGTGGACTACCTGGTGCCTCTAG-3'	5'-CGCCTCTGTCATTCGTGCTTCC-3'
human GAPDH	5'-CTTTGGTATCGTGGAAGGACTC-3'	5'-GTAGAGGCAGGGATGATGTTCT-3'

Table S2. The antibodies used in the study

Abbreviation	Antibody full name	Manufacture	Product No.	Concentration	Application
Periostin	Periostin (H-300)	santa cruz	sc-67233	1:1000	WB
CD163	anti-CD163 antibody	abcam	Ab182422	1:1000	WB
p-Erk	Phospho-p44/42 MAPK (Erk1/2)	CST	#4370	1:1000	WB
Erk	p44/42 MAPK (Erk1/2) (137F5)	CST	#4695	1:1000	WB
GAPDH	GAPDH	Zen	200306-7E4	1:5000	WB
Periostin	Periostin (H-300)	santa cruz	sc-67233	1:200	IF
CD68	anti-CD68 antibody	abcam	ab201340	1:200	IHC
CD163	anti-CD163 antibody	abcam	Ab182422	1:200	IHC
NOS2	NOS2	santa cruz	sc-7271	1:200	IHC
CD163	Alexa Fluor 647 Mouse Anti-Human CD163	BD	562669	5μl/test	Flow Cytometry
NOS2	NOS2	santa cruz	sc-7271	5μl/test	Flow Cytometry
Integrin αM	FITC Rat Anti-CD11b	BD	557396	2μl/test	Flow Cytometry

Table S3. The inclusion and exclusion criteria in the animal experiment.

inclusion criteria	exclusion criteria
**1. Male.**<br>**2. Body initial weight 200-250g.**<br>**3. General healthy.**<br>**4. Without any oral disease or developmental abnormalities.**	**1. Animals die before the end of the experiment.**<br>**2. Post-operative infection.**<br>**3. Other systemic disease (whether related to surgery) after surgery.**

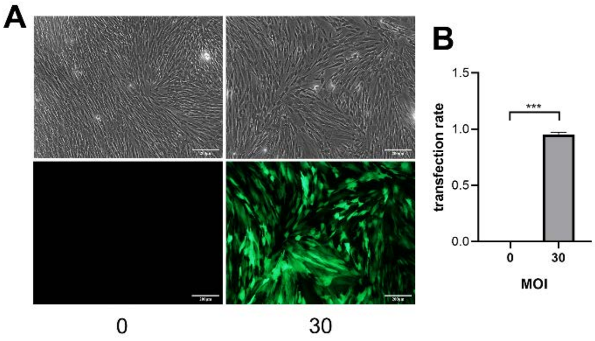

Fig. S1 Transfection of DFSCs.

(A) Representative images and analysis (B) of the lentiviruses transfection rate of DFSCs. Cells in the upper and lower columns were observed under bright-field and fluorescence field microscopy, respectively. MOI: multiplicity of infection. $*p < 0.05$; $**p < 0.01$; $***p < 0.001$. Error bars are means ± SD. Data were analyzed using independent unpaired two-tailed Student's t-test.
